# The Rho Guanine Nucleotide Exchange Factor DRhoGEF2 Is a Genetic Modifier of the PI3K Pathway in *Drosophila*

**DOI:** 10.1371/journal.pone.0152259

**Published:** 2016-03-25

**Authors:** Ying-Ju Chang, Lily Zhou, Richard Binari, Armen Manoukian, Tak Mak, Helen McNeill, Vuk Stambolic

**Affiliations:** 1 Princess Margaret Cancer Center/University Health Network, Toronto, Ontario, Canada; 2 Department of Medical Biophysics, University of Toronto, Toronto, Canada; 3 Lunenfeld-Tanenbaum Research Institute/Mount Sinai Hospital, Toronto, Ontario, Canada; 4 Department of Molecular Genetics, University of Toronto, Toronto, Canada; Simon Fraser University, CANADA

## Abstract

The insulin/IGF-1 signaling pathway mediates various physiological processes associated with human health. Components of this pathway are highly conserved throughout eukaryotic evolution. In *Drosophila*, the PTEN ortholog and its mammalian counterpart downregulate insulin/IGF signaling by antagonizing the PI3-kinase function. From a dominant loss-of-function genetic screen, we discovered that mutations of a Dbl-family member, the guanine nucleotide exchange factor DRhoGEF2 (*DRhoGEF2*^*2(l)04291*^), suppressed the *PTEN*-overexpression eye phenotype. dAkt/dPKB phosphorylation, a measure of PI3K signaling pathway activation, increased in the eye discs from the heterozygous DRhoGEF2 wandering third instar larvae. Overexpression of DRhoGEF2, and it’s functional mammalian ortholog PDZ-RhoGEF (ArhGEF11), at various stages of eye development, resulted in both dPKB/Akt-dependent and -independent phenotypes, reflecting the complexity in the crosstalk between PI3K and Rho signaling in *Drosophila*.

## Introduction

In higher eukaryotes, the Insulin/IGF-1 signaling pathway plays a key role in control of growth, development and differentiation, metabolic homeostasis and aging, acting via the insulin receptor (IR) and the insulin-like growth factor receptor (IGF-1R) [1–4] Briefly, ligand-activated IR and IGF-1R phosphorylates IRSs at tyrosine residues and thereby recruits various SH2-containing signaling proteins, including p85 (the regulatory subunit of PI3-kinase), growth factor receptor bound protein 2 (Grb2), SH2-containing phosphatase-2, (SHP), isoforms of SH2-containing protein (Shc), and c-Cbl-associated protein (CAP), to transduce insulin or IGF-1 action. Via these distinct adaptor molecules, insulin/IGF-1 signaling triggers signaling cascades that are initiated by PI3-kinase, small GTPase Ras, and c-Cbl [1, 5–8]. Among all the adaptor proteins, IRS-1 and IRS-2 are the common elements in transmitting the signals from ligand-activated IR and IGF-1R to activate PI3-kinase/PKB/Akt signaling [9–12].

The components of this pathway are highly conserved throughout eukaryotic evolution. In *Drosophila*, a PTEN ortholog and its mammalian counterpart negatively regulate insulin/IGF signaling by antagonizing PI3-kinase function. PTEN (phosphatase and tensin homology on chromosome 10) is frequently deleted in advanced human cancers. Germ line loss of PTEN is directly linked to the development of the PTEN hamartoma tumor syndrome (PHTS), a predisposition for the development of benign tumors in various organs [13]. Somatic PTEN mutations, mostly leading to complete loss of PTEN function, are found in a wide variety of human cancers [14]. Moreover, PTEN heterozygosity may be sufficient in promoting tumorigenesis in certain cellular contexts [15]. It is well established that PTEN mechanistically functions as a PIP3 (phophatidylinositol-3,4,5-triphosphate) 3’-phosphatase to reduce the level of intracellular PIP3, which antagonizes phosphoinositide 3-kinase (PI3K) [16, 17]. PIP3 recruits phosphoinositide-dependent protein kinase 1 (PDK1) and protein kinase B/mouse leukemia virus Akt 8 (PKB/Akt) to the cytoplasmic membrane where PDK1 and mammalian target for rapamycin complex 2 (mTORC2) activate PKB/Akt [18, 19]. By antagonizing PI3K-PKB/Akt, PTEN represses cell proliferation through induction of apoptosis and/or cell cycle arrest [20, 21]. Acting within an evolutionarily conserved cascade, PTEN also participates in the control of cell size, aging, polarity, and migration [15, 22–25]. In addition to the genetic loss of function, many cancers feature loss of PTEN expression by promoter methylation [26–28]. PTEN is also subjected to extensive regulatory post-translational modifications [27–29].

Conserved PTEN function has been characterized in a tissue-specific or cell-type specific fashion in both *Drosophila* compound eye and various tissues in mice [23, 30]. We performed a genetic screen searching for genes that can modify PTEN function. Disruption of DRhoGEF2, a member of the Rho-GEF family, partially rescued the small eye phenotype elicited by PTEN-overexpression [31, 32]. DRhoGEF2/Rho1 signaling affected the activity of dPKB/dAkt, an effector in the PI3K signaling pathway, during eye development. Our findings indicate that the balanced control of PI3K signaling, including the inputs from DRhoGEF2/Rho1, is necessary for the integrity of the *Drosophila* compound eye.

## Materials and Methods

### Fly stocks and husbandry

The PTEN overexpression transgenic fly line (*w*^*+*^*;GMR-GAL4>UAS-PTEN/*CyO) was generated in our lab as described previously [33]. The *P*-element line for *DRhoGEF2* (*cn*^*1*^*PRhoGEF2*^*04291*^*/CyO;ry*^*506*^, stock number 11369 *and w*^*1118*^*;P{RB}DRhoGEF2*^*e03784*^, stock number 18190), the driver lines, *GMR-GAL4/II*, *EYE-GAL4/II*, and EMS (Ethylmethanesulfonate) Rho1 mutant line (Rho1^E3.10^), (*w*^*a*^*N*^*fa-g*^*;Rho1*^*E3*.*10*^*/CyO*, stock number 3167) [34], *Drosophila* Rho kinase mutant line, Drok^2^ (*rok*^*2*^*/FM7*, *stock number 6665)* [35, 36], and a *P*-element enhancer line of RhoGAPp190 (*RhoGAPp190*^*EY08765*^) (*y1w*^*67c23*^*P{EPgy2}RhoGAPp190*^*EY08765*^, stock number 20177) and several RNAi mutant lines were obtained from Bloomington *Drosophila* Stock Center at Indiana University: GFP^RNAi^ (y[1] sc[*] v[1]; P{y[+t7.7] v[+t1.8] = VALIUM20-EGFP.shRNA.3}attP2, stock number 41560), DRhoGEF2^RNAi^ (*y1v1;P{TRiP*.*JF01747}attP2*, stock number 31239), and Rho1^RNAi^ (*y*^*1*^sc*v^*1*^*;P{TRiP*.*HMS00375}attP2/TM3*,*Sb1*, stock number 32383). Two EMS-induced mutant lines *DRhoGEF2*^*3w18*^*/CyO* and *DRhoGEF2*^*4*.*1*^*/CyO* were kindly provided by Dr. Armen Manoukian, Department of Medical Biophysics, University of Toronto (originally generated from Dr. Norbert Perrimon’s Laboratory at Harvard University) [31]. Canton-S, *w+;+/+;ry*^*506*^, and *w*^*1118*^ were used as wild type controls. Stocks were maintained and all experiments were conducted at 25°C on a 12h:12h light:dark cycle at constant humidity using standard sugar/yeast/agar (SYA) medium.

### Transgene constructs and germline transformation

The 8.6 kb full-length DNA of *DRhoGEF2* was cloned from a *Drosophila melanogaster* BAC clone containing *DRhoGEF2* cDNA obtained from Research Genetics, subcloned into *pUAST* [33] and used to generate the *pUAST-DRhoGEF2*, *pUAST-PDZ-RhoGEF*, and *pUAST-PDZ-RhoGEF*^*d8*^ transgenenic line by injection into *w*^*1118*^ embryos for germ line transformation as described previously [37]. Three *DRhoGEF2* transgenic lines were generated (*w;UAS-DRhoGEF2/CyO*, *w;UAS-DRhoGEF2/TM3*, *and w;UAS-DRhoGEF2/FM7*).

### Lethality rescue experiment

A ARM-GAL4 binary system was used to express transgene: *w*^*+*^*;UAS-DRhoGEF2/CyO* or *w*^*+*^*;UAS-mycPDZ-RhoGEF/CyO* in the fly in the presence of a *P*-element insertion mutant allele of *DRhoGEF2*, *w*^*+*^*;DRhoGEF2*^*04291*^*/CyO* and chemically induced point mutation *w*^*+*^*;DRhoGEF2*^*3w18*^*/CyO*. Virgin females carrying *w*^*+*^*;DRhoGEF2*^*04291*^*/CyO;ARM-GAL4/TM3* were crossed to males carrying *w*^*+*^*;DRhoGEF2*^*3w18*^*/CyO;UAS-DRhoGEF2*^*wt*^*/TM3*, *w*^*+*^*;DRhoGEF2*^*3w18*^*/CyO;UAS-mycPDZ-RhoGEF/TM3*, *or w*^*+*^*;DRhoGEF2*^*3w18*^*/CyO;UAS-mycPDZ-RhoGEF*^*d8*^*/TM3*. The genotype of F1 flies; *w*^*+*^*;DRhoGEF2*^*04291*^*/DRhoGEF2*^*3w18*^*;ARM-GAL4/UAS-DRhoGEF2*^*wt*^, *w*^*+*^*;DRhoGEF2*^*04291*^*/DRhoGEF2*^*3w18*^*;ARM-GAL4/UAS-mycPDZ-RhoGEF*, and *w*^*+*^*;DRhoGEF2*^*04291*^*/DRhoGEF2*^*3w18*^*;ARM-GAL4/UAS-flagPDZ-RhoGEF*^*d8*^ were assayed for viable adult flies. At least 2,000 flies were scored.

### Genetic crosses

Standard genetic crosses were set up for ectopic expression of DRhoGEF2 in the fly eyes. DRhoGEF2 was overexpressed in the specific stage of eye development using the upstream activation sequence (UAS)-GAL4 binary system [33]. During eye development, *GMR-GAL4* (glass multiple reporter driven *GAL4* expression) was employed to drive expression in the R cells in the eye imaginal disc and *ey-GAL4* (eyeless promoter driven *GAL4* expression) was used to overexpress the transgenes in the anterior, undifferentiated region of the eye imaginal disc during the third instar larval stage [38].

### Ommatidial structure

*Drosophila* eyes were fixed in 2% osmium/1% glutaraldehyde/0.1 M phosphate buffer (pH 7.2) for 30 min and followed by one change with fresh 2% osmium. After washing with 0.1 M phosphate buffer, osmiums fixed eyes were dehydrated with ethanol and ethanol was replaced by propylene oxide. Eyes were embedded in Durcapan resin mixture (epoxy resin, hardener, accelerator, and plasticizer) in the modules for sectioning. Sections were stained with 1% toluidine blue solution.

### Immunohistochemical analysis for apoptosis and cell fate determination

The eye imaginal discs were dissected from the third-instar larvae in S2 insect medium. Apoptosis was determined by staining with 3 mg/ml of acridine orange (Sigma-Aldrich). For cell proliferation, dissected discs were labeled with BrdU (bromodeoxyruidine, Becton Dickson) as described [39]. Briefly, BrdU labeled eye discs fixed in PBS/4% paraformaldehyde (PFA), were denatured by HCl, and neutralized by PBS. Apoptosis was analyzed with a Zeiss fluorescent microscope. In order to generate gain-of-function clones, the FLP-out GAL4 system (flipase driven GAL4 expression) was employed [40]. In brief, virgin females *hsflp;act>y*^*+*^*>GAL4UASGFP/CyO* were crossed with *w+;UAS-DRhoGEF2/UAS-DRhoGEF2* or *w+;UAS-mycPDZ-RhoGEF/UAS-mycPDZ-RhoGEF* at 18°C for 3 days, then, parental flies were flipped out. Embryos were heat shocked for 45 min at 37°C and maintained at 25°C. Eye imaginal discs from wandering third-instar larvae were dissected and fixed in PBS/4% PFA (Sigma-Aldrich), washed in PBS/0.1% Triton X-100 (Sigma-Aldrich), and incubated overnight with primary antibody. Discs were stained with rat anti-Elav (Developmental Studies Hybridoma Bank, University of Iowa), goat-anti-rat-Cy5 (Jackson Lab), and phalloidin-rhodamine (Molecular Probe). The stained discs were analyzed with a Zeiss confocal microscope.

### Phenotypic and mosaic analysis of adult eyes

All adult eye phenotypes were analyzed in females raised at 25°C unless indicated otherwise. The external eye phenotype was analyzed using a standard protocol for scanning electronic microscopy. For ommatidial organization, transverse sections were prepared for light and transmission electron microscopy.

### Immunoblotting

To prepare total protein lysates, five to 10 eye imaginal discs were homogenized in cell lysis buffer (20 mM Tris (pH7.5), 150 mM NaCl, 1 mM EDTA, 1 mM EGTA, 1% Triton x-100, phosphatase inhibitors (2.5 mM sodium pyrophosphate, 1 mM β-glycerophosphate, 1 mM sodium orthovanadate), and protease inhibitors (1 mg/ml leupeptin, 1mM phenylmethanesulfonyl fluoride (PMSF)). Phosphorylation of dPKB/dAkt (serine 505), total dPKB/dAkt, and β-tubulin were detected using antibodies for phospho-S505 of dPKB/dAkt and total dPKB/dAkt (Cell Signaling Technology), β-tubulin (Upstate).

## Results

### DRhoGEF2^2(l)04291^ suppresses PTEN overexpression-induced developmental eye defects

**Fig 1:**
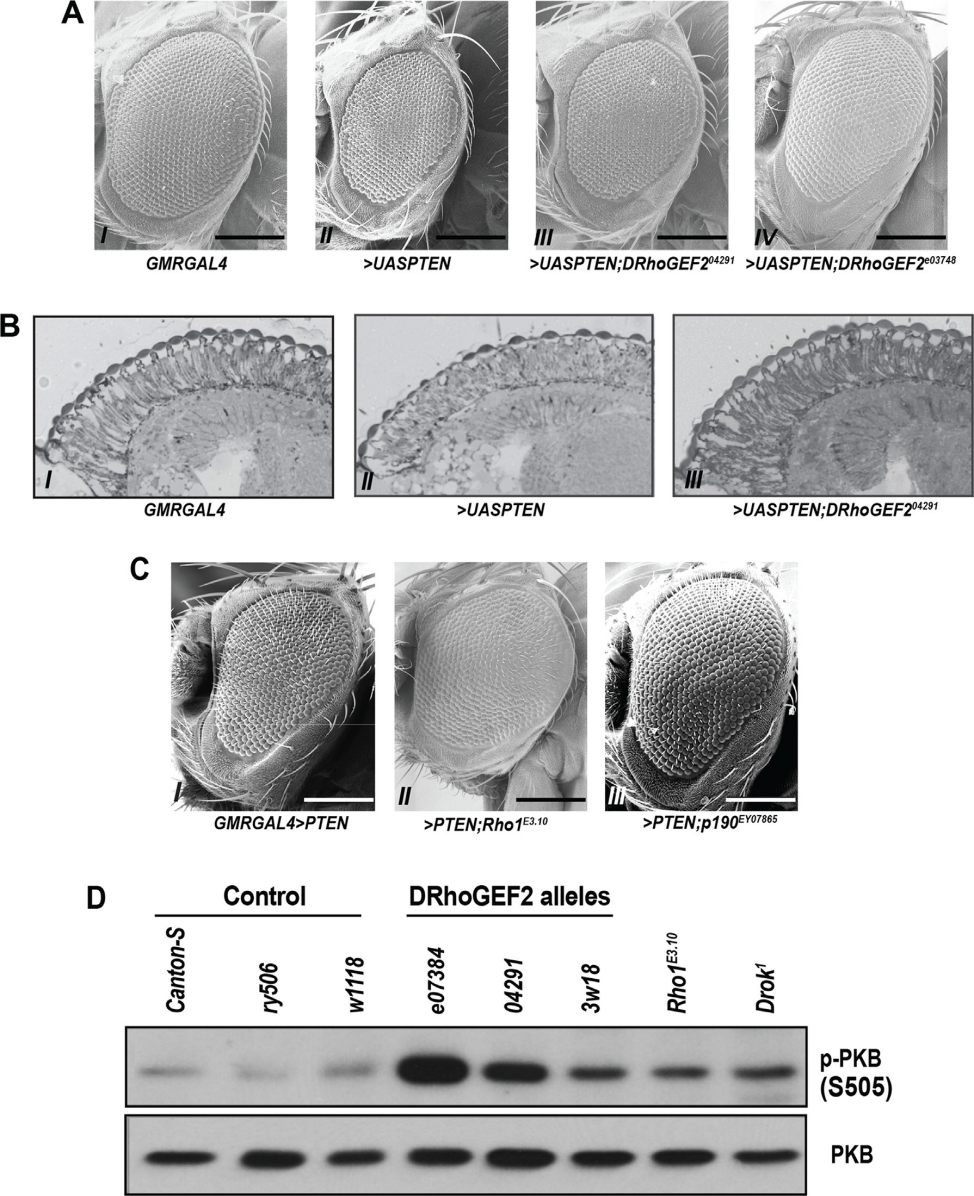
Rho signaling suppresses the PTEN-overexpression eye phenotype via dPKB/dAkt activation. (A) Scanning electronic micrograph of adult eyes from (I) *GMR-GAL4/+*, (II) *GMR-GAL4>UAS-PTEN/CyO*, (III) *GMR-GAL4>UAS-PTEN/DRhoGEF2*^*04291*^, and (IV) *GMR-GAL4>UAS-PTEN/DRhoGEF2*^*e03784*^. Scale bar = 200 μm. (B) Toluidine blue-stained longitudinal retinal sections of adult eyes from (I) *GMR-GAL4/+* and (II) *GMR-GAL4>UAS-PTEN* and (III) *GMR-GAL4>UAS-PTEN/DRhoGEF2*^*04291*^. (C) Scanning electronic micrograph of adult eyes from (I) *GMR-GAL>UAS-PTEN* and (II) *GMR-GAL4>UASPTEN/Rho*^*E3*.*10*^. Scale bar = 200 m. μ(D) Scanning electronic micrograph of adult eyes from (I) *GMR-GAL4>UAS-PTEN* and (II) *w67c23P{EPgy2}RhoGAPp190EY08765/+;GMR-GAL4>UAS-PTEN/+*. Scale bar = 200 μm. (D) dPKB/dAkt phosphorylation in the 3^rd^ instar larval eye discs from the wild type controls (*Canton-S*; *ry*^*506*^, and *w1118*) and the mutants (*DRhoGEF2*^*04291*^*/CyO*, *DRhoGEF2*^*e0378*^*/CyO*, *Rho1*^*E3*.*10*^*/CyO*, and *Drok*^*1*^*/FM7*), representative of three independent experiments.

We performed a dominant modifier screen for mutations that affect the small eye phenotype resulting from PTEN overexpression, by crossing flies with GMR-GAL4-driven *PTEN* expression to a collection of 1045 *P*-element strains. Each strain comprises a single *P*-element insertion in one allele of each gene, which when homozygous leads to embryonic lethality [41]. Changes in the eye size of F1 progenies were scored for suppressors or enhancers of the small eye phenotype. One of the *P*-element insertions, *I(2)04291*, which maps to 53F01-2 cytological location on the right arm of chromosome 2, partially rescued the PTEN-driven small eye phenotype (Fig 1A-II, IA-III). *I(2)04291* inserts at the 5’-end of the promoter region of *DRhoGEF2* and disrupts its expression (*DRhoGEF2*^*04291*^) [32]. The interaction between DRhoGEF2 and PTEN was further verified using another piggyBac-based *P*-element insertion line in the same gene, *DRhoGEF2*^*e03784*^ (Fig 1A-IV) and *DRhoGEF2*^*3w18*^, one of the chemically induced alleles from the *DRhoGEF2*^*04291*^complementation group [31, 32] (S1A–II Fig), as well as the *DRhoGEF2 RNAi* (S1A–II Fig). To investigate the internal morphology underlying the difference, eye sections were examined, revealing that the mutant *DRhoGEF2* alleles suppressed the PTEN-overexpression defects in retinal cell elongation (Fig 1B) without affecting the number of ommatidia (S1B Fig).

Further indicative of a functional interaction of Rho signaling with PTEN, introduction of a mutant allele of *Rho1*^*E3*.*10*^, an effector of DRhoGEF2, suppressed the small and the flattened appearance eye phenotype resulting from PTEN overexpression (Fig 1C-II). Moreover, a similar phenotype was also observed when the Rho1 activity was impaired by either overexpression of RhoGAPp190 (*RhoGAPp190*^*EY08765*^, *p190*^*EY08765*^) (Fig 1C-III) or upon *Rho1 RNAi* (*Rho1*^*RNAi*^) (S1C–II Fig).

Consistent with the function of PTEN in opposing the PI3K pathway, overexpression of PTEN affected both eye thickness and size, phenotypic features previously linked to the role of PI3K in eye development [42] (Fig 1A-II). In line with this, activation-specific phosphorylation of dPKB/dAkt, an effector of PI3K signals, was increased at serine 505 (S505), a residue homologous to mammalian serine 473 (S473) of PKB/Akt, in the eye imaginal discs from the wandering third instar larvae of the *DRhoGEF2*^*04291*^ and *GMRGAL4>DRhoGEF2*^*RNAi*^ flies (Fig 1D, S1D Fig). Similarly, eye imaginal discs with a mutant allele of *Rho1 (Rho1*^*E3*.*10*^) and *Drok (Drok*^*1*^), the downstream effectors of DRhoGEF2, also displayed elevated dPKB/dAkt S505 phosphorylation (Fig 1D).

### Identification the mammalian ortholog of DRhoGEF2

Alignment of the amino acid sequences of mammalian Rho-GEFs with DRhoGEF2, identifies PDZ-RhoGEF as its closest mammalian counterpart (S2A Fig). To functionally explore this, genetic complementation was performed using flies carrying the *PDZ-RhoGEF* or the *DRhoGEF2* transgene. Expression of *PDZ-RhoGEF* or *DRhoGEF2*, but not the alternative spliced isoform of *PDZ-RhoGEF* (*PDZ-RhoGEF*^*d8*^), under the control of the *armadillo-GAL4* (*ARM-GAL4*) system driving transgene expression during early embryo development, rescued the lethality caused by the homozygous *DRhoGEF2*^*04291*^ (*DRhoGEF2*^*04291*^/*DRhoGEF2*^*04291*^) or the heterozygous *DRhoGEF2*^*04291*^ with the EMS allele *DRhoGEF2*^*3w18*^ (*DRhoGEF2*^*04291*^/*DRhoGEF2*^*3w18*^) (Table 1). Of note, certain wild type embryos with either transgene overexpression died at late 2^nd^ or early 3^rd^ instar larval stage with growth retardation (S2B Fig), resulting in a decrease in the total number of rescued adult flies (Table 1).

**Table 1:** The lethality rescue of DRhoGE2 homozygous mutant alleles by DRhoGEF2 and its mammalian orthologs.

Genotype of viable adults	Viable adults
*DRhoGEF2*^*04291*^,*DRhoGEF2*^*3w18*^*;ARM-GAL4>UAS-DRhoGEF2*	51 (153)
*DRhoGEF2*^*04291*^,*DRhoGEF2*^*3w18*^*;ARM-GAL4>UAS-mycPDZ-RhoGEF*	35 (125)
*DRhoGEF2*^*04291*^,*DRhoGEF2*^*3w18*^*;ARM-GAL4>UAS-flagPDZ-RhoGEF*^*d8*^	0 (125)

The lethality rescue was calculated as percent of viable flies of each genotype of total vial adult flies. Rescue by DRhoGE2 transgene, total 2455 viable flies were scored. Rescue by PDZ-RhoGEF and PDZ-RhoGEF^d8^ transgenes, total 2000 viable flies were scored. Numbers in parentheses indicate expected numbers relative to total number of viable flies based on Mendelian frequency.

### Optimal DRhoGEF2 expression is required for neuronal precursor cell survival

**Fig 2:**
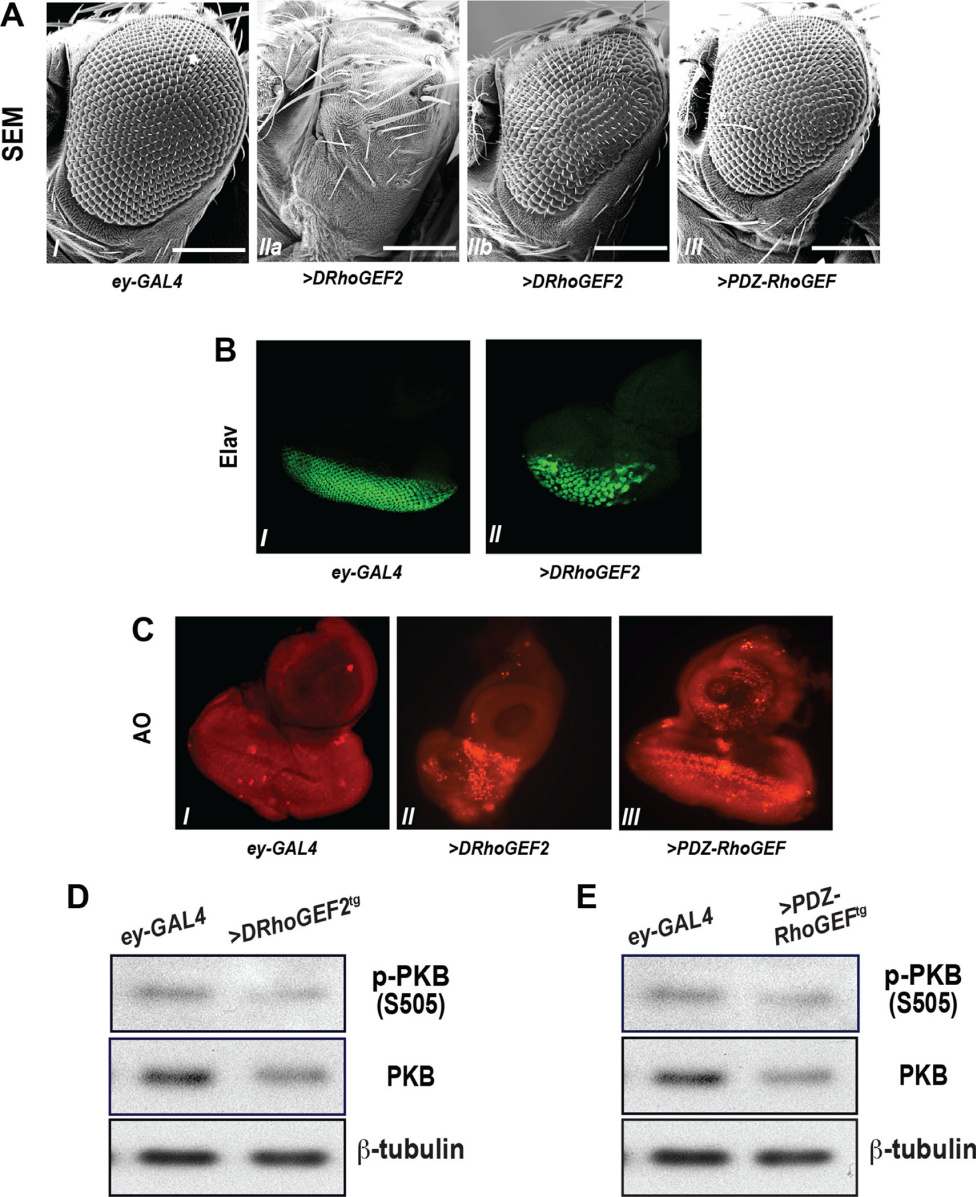
The small eye phenotype elicited by *ey-GAL4*-driven *DRhoGEF2*/*PDZ-RhoGEF* expression. (A) Scanning electronic micrographs of adult eyes with ectopic expression of *DRhoGEF2* or *PDZ-RhoGEF* under the control of *ey-GAL4*. (I) *+/+; ey-GAL4/+*, (IIa,IIb) variable small eye phenotype with *UAS-DRhoGEF2/+;ey-GAL4/+*, and (III) *UAS-mycPDZ-RhoGEF/+; ey-GAL4/+*. Scale bar = 200 μm. (B) Disorganized neuronal cell clusters upon *ey-GAL4>DRhoGEF2* overexpression. (I) *+/+;ey-GAL4/+* and (II) *w+;UAS-DRhoGEF2/+; ey-GAL4/+*. (C) Detection of apoptosis by acridine orange (AO) staining in the 3^rd^ instar eye disc with *DRhoGEF2* or *PDZ-RhoGEF* overexpression under the control of (I) *+/+;ey-GAL4/+*, (II) *UAS-DRhoGEF2/+;ey-GAL4/+*, and (III) *UAS-mycPDZ-RhoGEF/+; ey-GAL4/+*. (D) & (E) Phosphorylation of dPKB/dAkt in the 3^rd^ instar larval eye imaginal discs from *+/+;ey-GAL4/+* (*ey-GAL4*) and *UAS-DRhoGEF2/+;ey-GAL4/+* (>*DRhoGEF2*^*tg*^) (D) or *UAS-mycPDZ-RhoGEF; ey-GAL4/+* (>*PDZ-RhoGEF*^*tg*^) (E).

To determine the effect of Rho signaling on eye development, the expression of *DRhoGEF2* or *PDZ-RhoGEF* was placed under the control of *eyeless-GAL4* (*ey-GAL4*), resulting in expression in the neuronal precursor cells at the anterior of the morphorgentic furrow (MF). DRhoGEF2 overexpression led to severe eye damage, small or no eye phenotype (Fig 2A-IIa and 2A-IIb), whereas overexpression of PDZ-RhoGEF resulted in a less severe reduced eye size phenotype (Fig 2A-III). Staining of eye imaginal discs from the wandering 3^rd^ instar larvae with an antibody for Elav, a neuron specific transcription factor, revealed disorganized neuronal cell clusters (Fig 2B-II). An increase in acridine orange (AO) positive cells upon transgene expression indicative of apoptosis (Fig 2C-II and 2C-III) was accompanied by reduced dPKB/dAkt S505 phosphorylation and the total protein levels of dPKB/dAkt (Fig 2D and 2E).

### Elevated Rho signaling disrupts photoreceptor structure

Ectopic expression of *DRhoGEF2/PDZ-RhoGEF* in the post-mitotic cells that is posterior to the MF by GMR-GAL4 disrupted the outer ommatidial lattice and led to loss of bristles resulting in a rough eye phenotype and reduced eye size (S3A–II and S3A–III Fig). To further characterize the cellular abnormalities in the rough eyes, toluidine stained transverse sections of the adult compound eyes were analyzed by light microscopy. GMR-GAL4-driven overexpression of *DRhoGEF2/PDZ-RhoGEF* disrupted the organization of the ommatidial lattice of the adult eye with noticeable vesicles containing rhabdomere remnants, indicative of the defective photoreceptor and accessory cell pattern formation (S3B–II Fig, S3B–III Fig). Interestingly, judging by AO staining and BrdU uptake of the 3^rd^ instar eye discs, respectively, there was no difference in proliferation (S3C Fig), cell survival (S3D Fig) or dPKB/dAkt S505 phosphorylation between GMR-GAL4-driven *DRhoGEF2/PDZ-RhoGEF*-overexpressing and control eye discs (S3E Fig and S3F Fig). Moreover, heat-shock (HS)-actin-GAL4-driven clonal overexpression of *DRhoGEF2/PDZ-RhoGEF* at earlier stages of eye development had no impact on the organization or the actin cytoskeleton (S4A Fig and S4B Fig). However, HS-induced clonal expression of *DRhoGEF2* or *PDZ-RhoGEF* in the differentiated eye cells also caused damage in the adult eyes (S4C Fig). Thus, these results suggest that ectopic expression of DRhoGEF2/PDZ-RhoGEF in the differentiated neuronal cells affects eye development at steps following cell fate determination. Considering that GMR-driven expression of Rho1 leads to a similar eye phenotype [43], we used a mutant line carrying *Rho1*^*E3*.*10*^ or GMR-GAL4-driven *RhoGAPp190* overexpression, respectively, to reduce Rho signaling throughput in DRhoGEF2/PDZ-RhoGEF-overexpressing adult eyes. Indeed, the damaged external eye structure of an adult fly eye resulting from GMR-GAL4 driven *DRhoGEF2* expression was partially rescued when Rho signaling was reduced (S4D Fig).

## Discussion

DRhoGEF2 is a *Drosophila* member of the Dbl family of Guanidine Exchange Factors (GEFs), which transmit Gɑ-protein coupled receptor (Fog/Cta)-dependent and -independent signals to Rho1, to regulate cell shape, invagination, and epithelial folding during embryogenesis and eye development [31, 32, 44–46]. Here, we show that *DRhoGEF2* and the *Drosophila* effector Rho1, genetically interact with PTEN. DRhoGEF2 loss of function increases dPKB/dAkt activity and suppresses the eye phenotype elicited by PTEN-overexpression, further connecting the Rho1 and PI3K pathways in the Drosophila eye. Importantly, DRhoGEF2 and human PDZ-RhoGEF are functionally redundant in maintaining ommatidia integrity.

The eye phenotype brought on by PTEN overexpression was suppressed by reduced Rho1 signaling, either via the partial loss of function mutants of DRhoGEF2 or it’s downstream effector, Rho1. Notably, activity of dPKB/dAkt was also elevated in *DRhoGEF2*^*04291*^ and *Rho*^*E3*.*10*^ eye discs with reduced Rho signaling (Fig 1D). Previous work has shown that PTEN overexpression affects *Drosophila* eye size by inhibiting cell cycle progression at early mitosis and by promoting cell death during eye development [30]. The loss of one allele of *DRhoGEF2* had no effect on total number of ommatidia when combined with PTEN overexpression, suggesting that DRhoGEF2 does not impact the apoptosis or the reduced cell proliferation induced by PTEN overexpression, raising the possibility that DRhoGEF2 and PTEN may interact to control retinal cell elongation. Indeed, the flattened retina caused by PTEN overexpression in differentiated neuronal cells was partially rescued in *DRhoGEF2*^*04291*^ animals (Fig 1B). Moreover, previous work has shown that the DRhoGEF2^04291^ allele also suppressed the Rho1 overexpression-induced rough eye phenotype by restoring retinal cell elongation [32] and that the catalytic subunit of *Drosophila* PI3K, Dp110 affects retinal elongation [42]. Together, these data demonstrate that Rho1 and its regulator, DRhoGEF2 interact with the PI3-kinase/PTEN signaling pathway to control retinal structure.

Loss-of-function mutations of the components of the insulin/IGF-1 pathway, including the insulin receptor (InR), chico (Drosophila Insulin Receptor Substrate (IRS)), PI3-kinase, and dPKB/dAkt, lead to reduced cell growth during Drosophila eye and wing development [47–50] and impaired cell survival during Drosophila embryogenesis [51]. In agreement with the PI3K-opposing function of PTEN, mutant clones deficient for PTEN generated in the early 1^st^ instar larvae display a proliferative advantage compared to wild type twin clones [52]. Analogous to their relationship in mammalian systems, dPKB/dAkt has been firmly placed downstream of PTEN and PI3K in the fly [19]. Our findings that the reduction of DRhoGEF2 expression led to an increase in dPKB/dAkt phosphorylation in the 3rd instar larval eye imaginal discs (Fig 1D and S1E Fig), and a decrease when DRhoGEF2 expression was elevated in neuronal precursor cells (Fig 2D and 2E), also place Rho signaling upstream of dPKB/dAkt. It has been shown that Rho-kinases (ROCKI/II), mammalian orthologs of Drok, regulates insulin/IGF-1 signaling by phosphorylating the insulin receptor substrate 1 (IRS-1) at serine residues [53, 54]. Our findings raise the possibility that the genetic interaction between Rho1 and PTEN/PI3K signaling pathways may be mediated by Drok and chico, equivalent to their relationship in mammals. Regulation of the actin cytoskeleton, a process impacted by both PI3K-PKB/Akt and Rho signaling [46, 55, 56], could also be a contributing factor to the observed phenotypes and reflect another point of crosstalk between these two signaling pathways.

ey-GAL4-driven DRhoGEF2 expression led to increased apoptosis in 3^rd^ instar larval eye imaginal eye discs (Fig 2C), accompanied by a reduction of dPKB/dAkt phosphorylation and total dPKB/dAkt protein levels (Fig 2D), factors predicted to reduce cell survival [57, 58]. Interestingly, GMR-GAL4 driven DRhoGEF2 expression in differentiated neuronal cells resulted in an externally and internally disrupted compound eye without any effect on cell fate determination or dPKB/dAkt protein levels and activation (S3 Fig, S4 Fig), exposing a likely dPKB/Akt-independent effects of DRhoGEF2 on eye development at steps following cell fate determination. These, possibly cell-context functions of DRhoGEF2 at the later stages of eye development require further investigation.

Taken together, using *Drosophila* as a model, our work uncovers an intricate relationship between PI3K and Rho1 signaling pathways. Considering the high degree of conservation of the components of both pathways amongst vertebrate species, it will be of interest to determine the extent of pathway communication in regulation of other processes and tissue organization and development in other species.

## Supporting Information

S1 Fig Rho signaling suppresses the *PTEN*-overexpression eye phenotype via its effects on dPKB/dAkt activation. (A) Scanning electronic micrographs of adult eyes from (I) *GMR-GAL4>UAS-PTEN/CyO*, (II) *GMR-GAL4>UAS-PTEN/DRhoGEF2*^*3w18*^, and (III) *GMR-GAL4>UAS-PTEN/+;GFP*^*RNAi*^*/+*, (IV) *GMRGAL4>UAS-PTEN;DRhoGEF2*^*RNAi*^*/+*. Scale bar = 200 μm. (B) The ommatidial number in individual flies was determined by scanning electronic micrographs (n = 10). (C) The levels of dPKB/dAkt phosphorylation in the 3^rd^ instar larval eye imaginal discs in *GMRGAL4/+;GFP*^*RNAi*^/+ and *GMRGAL4/+;Rho1*^*RNAi*^*/+* and quantified using ImageJ. (D) Scanning electronic micrographs of adult fly eyes from (I) *GMRGAL4>UAS-PTEN;GFP*^*RNAi*^*/+* and (II) *GMRGAL4>UAS-PTEN/+;DRhoGEF2*^*RNAi*^*/+*. Scale bar = 200μm.
(TIF)

S2 Fig PDZ-RhoGEF is the mammalian ortholog of DRhoGEF2. (A) An unrooted phylogenetic analysis based on the ClustlW alignment of the amino acid sequence of five members of RGS-RhoGEF subfamily. The phylogenetic tree demonstrated that PDZ-RhoGEF is the closest mammalian ortholog of DRhoGEF2. (B) Embryos with *ARMGAL4* driven *DRhoGEF2* or *PDZ-RhoGEF* overexpression exhibited growth retardation and died during late 2nd or early 3^rd^ instar larval stage.
(TIF)

S3 Fig The rough eye phenotype resulting from *GMR-GAL4*-driven *DRhoGEF2*/*PDZ-RhoGEF* expression. (A) Scanning electron micrographs of adult eye s with ectopic expression of *DRhoGEF*2 or myc*PDZ-RhoGEF* under the control of GMR-GAL4. (I) *GMR-GAL4/+*, (II) *GMR-GAL4/UAS-DRhoGEF2*, and (III) *GMR-GAL4/UAS-mycPDZ-RhoGEF*. Scale bar = 200 μm. (B) Toluidine blue-stained transverse sections of the adult eye with *DRhoGEF2* or *PDZ-RhoGEF* overexpression. (I) *GMR-GAL4/+*, (II) *GMR-GAL4/UAS-DRhoGEF2*, and (III) *GMR-GAL4/UAS-mycPDZ-RhoGEF*. (C) Acridine orange (AO) staining in the 3^rd^ instar larval eye imaginal discs with *DRhoGEF2* or myc*PDZ-RhoGEF* overexpression. (I) *GMR-GAL4/+*, (II) *GMR-GAL4/UAS-DRhoGEF2*, and (III) *GMR-GAL4/UAS-mycPDZ-RhoGEF*. (D) Cell proliferation in *DRhoGEF2*- or *PDZ-RhoGEF*-overexpressing 3^rd^ instar larval eye imaginal discs, determined by BrdU incorporation. (I) *GMR-GAL4/+*, (II) *GMR-GAL4/UAS-DRhoGEF2*, and (III) *GMR-GAL4/UAS-mycPDZ-RhoGEF*. (E) & (F) Phosphorylation of dPKB/dAkt in the 3^rd^ instar larval eye imaginal discs with *DRhoGEF2* (C) or *PDZ-RhoGEF* (D) overexpression.
(TIF)

S4 Fig Overexpression of DRhoGEF2 has no effect on cell fate determination. (A) & (B) Immunostaining of the post-mitotic neuronal cells with ectopic *DRhoGEF2* (A) or *PDZ-RhoGEF* (B) expression induced by heat shock through mitotic recombination. (C) Scanning electronic micrographs of adult eyes from heat-induced recombination and gene expression. (I) *hsflp;act*,*FRT*,*GAL4>UAS-GFP/UAS-DRhoGEF2* and (II) *hsflp;act*,*FRT*,*GAL4>UAS-GFP/UAS-mycPDZ-RhoGEF*. (D) Scanning electronic micrographs of adult fly eyes from *GMR-GAL4>UAS-DRhoGEF2/CyO* (I), *GMR-GAL4>UAS-DRhoGEF2/Rho*^*E3*.*10*^ (II), *w67c23P{EPgy2}RhoGAPp190EY08765/+;GMR-GAL4>UAS-DRhoGEF2* (III). Scale bar = 200 μm.
(TIF)
