# Correction: The Rho Guanine Nucleotide Exchange Factor DRhoGEF2 Is a Genetic Modifier of the PI3K Pathway in *Drosophila*

**DOI:** 10.1371/journal.pone.0252252

**Published:** 2021-05-20

**Authors:** Ying-Ju Chang, Lily Zhou, Richard Binari, Armen Manoukian, Tak Mak, Helen McNeill, Vuk Stambolic

Following the publication of this article [1] concerns were raised regarding similarities between the *ey-GAL4* results presented in Fig 2D and 2E. Similarly, concerns were raised regarding similarities between the GMR-GAL4 results presented in S3E and S3F Fig. The corresponding author explained that for each pair of figure panels (Fig 2D and 2E; S3E and S3F Fig) the results were obtained in the same blot experiment. Lanes from the same original blot image were spliced together to present pertinent results in each panel; the control data were reused in Fig 2D and 2E and in S3E and S3F Fig.

**Fig 2:**
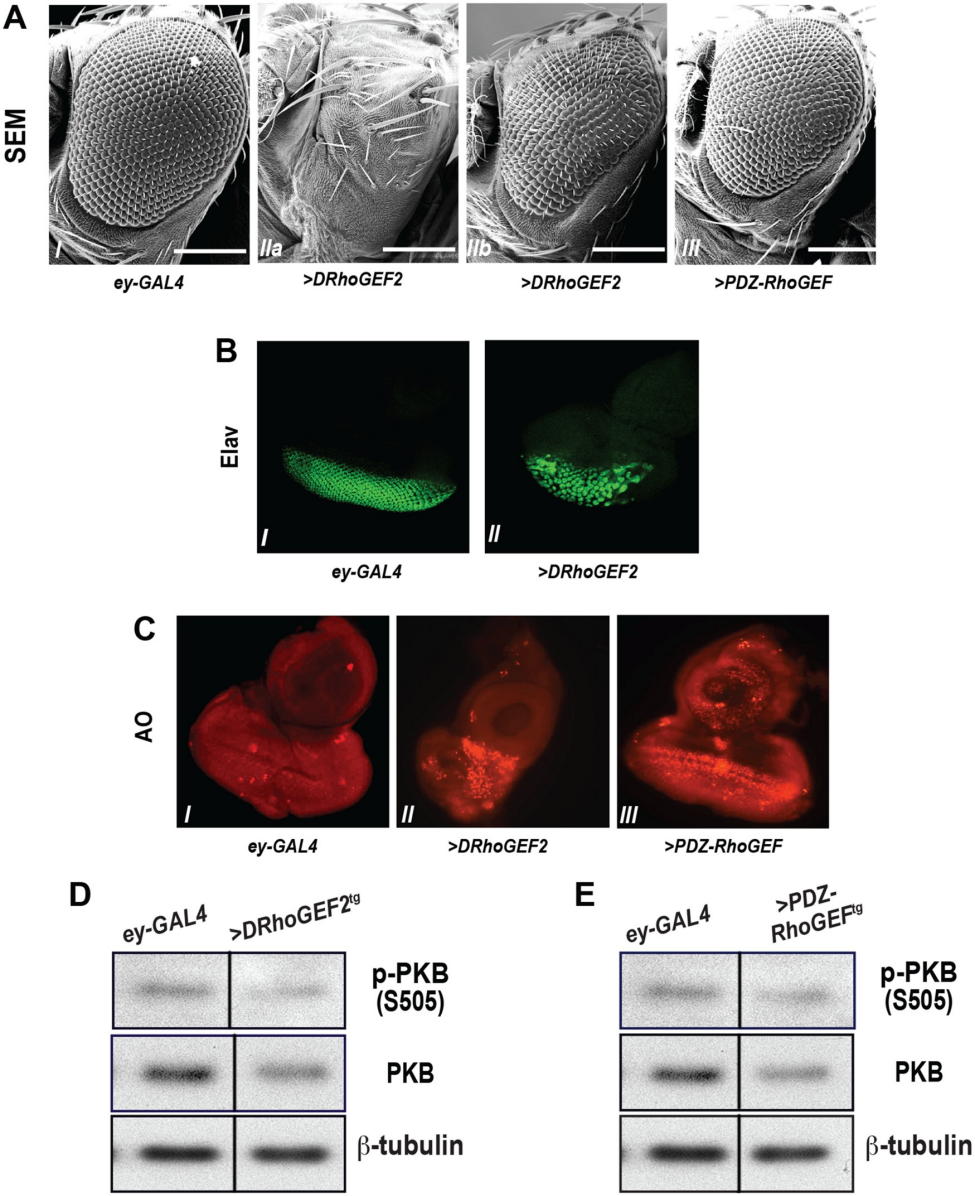
The small eye phenotype elicited by *ey-GAL4*-driven *DRhoGEF2*/*PDZ-RhoGEF* expression. (A) Scanning electronic micrographs of adult eyes with ectopic expression of *DRhoGEF2* or *PDZ-RhoGEF* under the control of *ey-GAL4*. (I) +/+; *ey-GAL4/+*, (IIa,IIb) variable small eye phenotype with *UAS-DRhoGEF2/+*;*ey-GAL4/+*, and (III) *UAS-mycPDZ-RhoGEF/+*; *ey-GAL4/+*. Scale bar = 200 μm. (B) Disorganized neuronal cell clusters upon *ey-GAL4>DRhoGEF2* overexpression. (I) +/+;*ey-GAL4/+* and (II) w+;*UAS-DRhoGEF2/+*; *ey-GAL4/+*. (C) Detection of apoptosis by acridine orange (AO) staining in the 3rd instar eye disc with *DRhoGEF2* or *PDZ-RhoGEF* overexpression under the control of (I) +/+;*ey-GAL4/+*, (II) *UAS-DRhoGEF2/+*;*ey-GAL4/+*, and (III) *UAS-mycPDZ-RhoGEF/+*; *ey-GAL4/+*. (D) & (E) Phosphorylation of dPKB/dAkt in the 3rd instar larval eye imaginal discs from +/+;*ey-GAL4/+* (*ey-GAL4*) (D) & (E) and *UAS-DRhoGEF2/+*;*ey-GAL4/+* (>*DRhoGEF2*^*tg*^) (D) or *UAS-mycPDZ-RhoGEF*; *ey-GAL4/+* (>*PDZ-RhoGEF*^*tg*^) (E). Results shown in panels D and E were obtained in the same western blot experiment for which underlying data are in S1 File of this Correction notice.

There was also an error in the final sentence of the S3 Fig legend, which referenced panels C and D instead of panels E and F as reporting the phosphorylation data.

The updated figure legends below clarify the duplicate use of the control results and address the referencing errors. S1 File presents the original blots underlying results shown in Fig 2D and 2E and S3E and S3F Fig.

## Supporting information

S1 File Original uncropped blots underlying results presented in Fig 2D and 2E, S3E and S3F Fig, with the S3 Fig results highlighted in red, and the Fig 2 results highlighted in blue. (TIF)

S3 Fig The rough eye phenotype resulting from *GMR-GAL4*-driven *DRhoGEF2/PDZ-RhoGEF* expression. Labels in (A-D) indicate samples with the following genotypes: (I) GMR-GAL4/+, (II) GMR-GAL4/UAS-*DRhoGEF2*, and (III) *GMR-GAL4/UAS-mycPDZ-RhoGEF*. (A) Scanning electron micrographs of adult eyes with ectopic expression of *DRhoGEF2* or *mycPDZ-RhoGEF* under the control of *GMR-GAL4*. Scale bar = 200 μm. (B) Toluidine blue-stained transverse sections of the adult eye with *DRhoGEF2* or *PDZ-RhoGEF* overexpression. (C) Acridine orange (AO) staining in the 3^rd^ instar larval eye imaginal discs with *DRhoGEF2* or *mycPDZ-RhoGEF* overexpression. (D) Cell proliferation in *DRhoGEF2-* or *PDZ-RhoGEF*-overexpressing 3^rd^ instar larval eye imaginal discs, determined by BrdU incorporation. (E, F) Phosphorylation of dPKB/dAkt in the 3^rd^ instar larval eye imaginal discs of control *GMR-GAL4* flies (E, F) or flies overexpressing *DRhoGEF2* (E) or *PDZ-RhoGEF* (F). Results shown in panels E and F were obtained in the same western blot experiment for which underlying data are in S1 File of this Correction notice.
(TIF)
