# Supplementary figures and images for: The Rho Guanine Nucleotide Exchange Factor DRhoGEF2 Is a Genetic Modifier of the PI3K Pathway in Drosophila
Source: PLoS One. 2016 Mar 25;11(3):e0152259. doi: 10.1371/journal.pone.0152259 (PMC4807833; doi:10.1371/journal.pone.0152259)

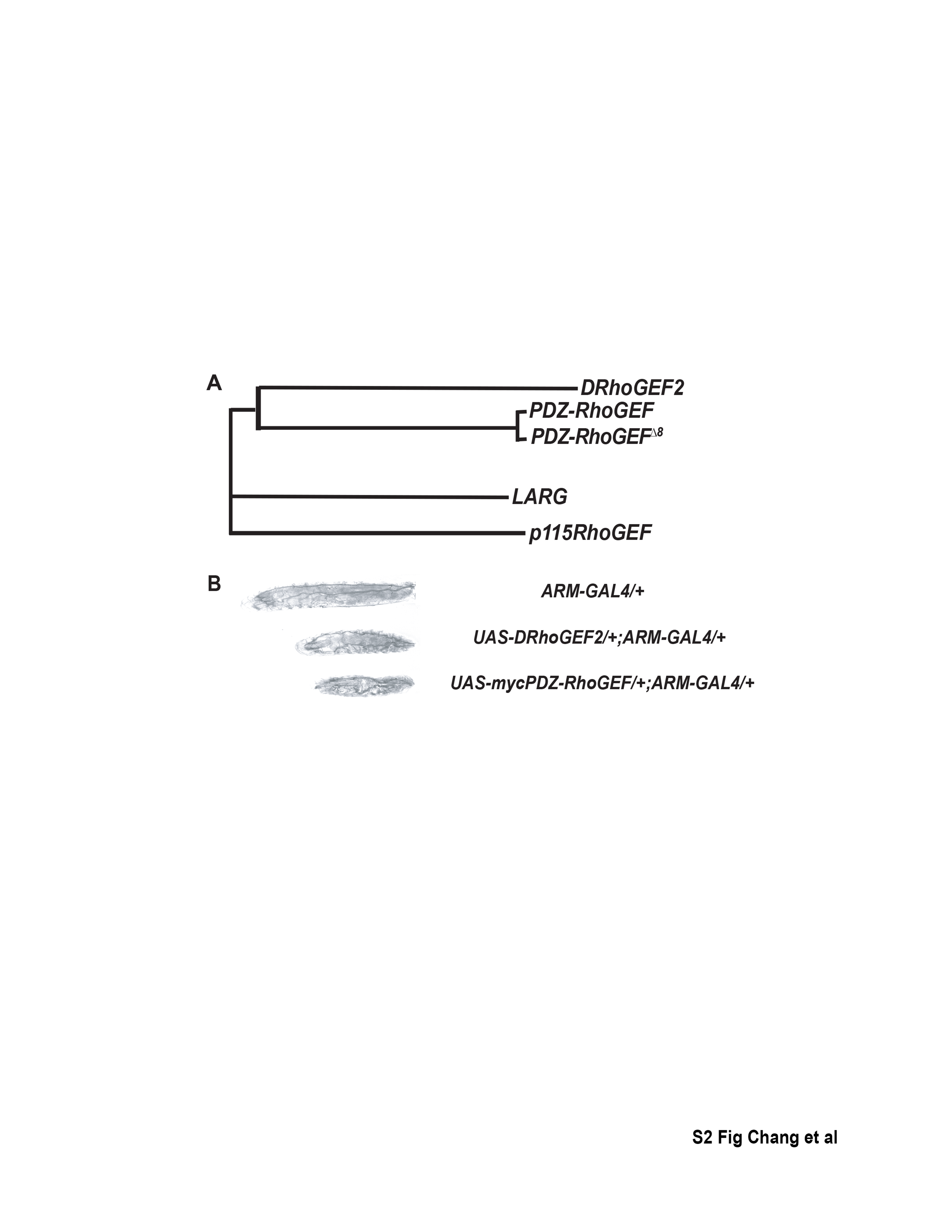

Supplement: S2 Fig — (A) An unrooted phylogenetic analysis based on the ClustlW alignment of the amino acid sequence of five members of RGS-RhoGEF subfamily. The phylogenetic tree demonstrated that PDZ-RhoGEF is the closest mammalian ortholog of DRhoGEF2. (B) Embryos with ARMGAL4 driven DRhoGEF2 or PDZ-RhoGEF overexpression exhibited growth retardation and died during late 2nd or early 3rd instar larval stage. (TIF) [file pone.0152259.s002.tif]

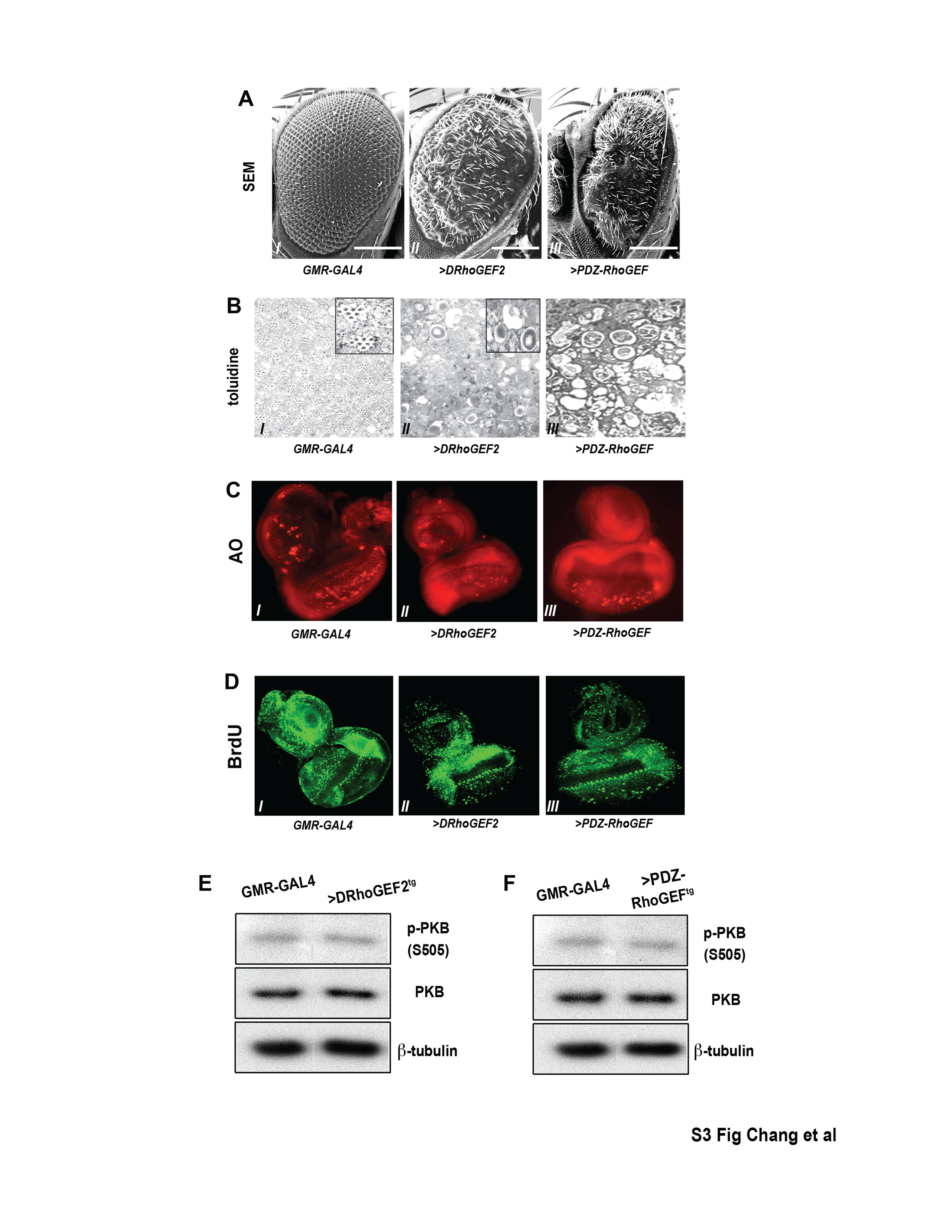

Supplement: S3 Fig — (A) Scanning electron micrographs of adult eye s with ectopic expression of DRhoGEF2 or mycPDZ-RhoGEF under the control of GMR-GAL4. (I) GMR-GAL4/+, (II) GMR-GAL4/UAS-DRhoGEF2, and (III) GMR-GAL4/UAS-mycPDZ-RhoGEF. Scale bar = 200 μm. (B) Toluidine blue-stained transverse sections of the adult eye with DRhoGEF2 or PDZ-RhoGEF overexpression. (I) GMR-GAL4/+, (II) GMR-GAL4/UAS-DRhoGEF2, and (III) GMR-GAL4/UAS-mycPDZ-RhoGEF. (C) Acridine orange (AO) staining in the 3rd instar larval eye imaginal discs with DRhoGEF2 or mycPDZ-RhoGEF overexpression. (I) GMR-GAL4/+, (II) GMR-GAL4/UAS-DRhoGEF2, and (III) GMR-GAL4/UAS-mycPDZ-RhoGEF. (D) Cell proliferation in DRhoGEF2- or PDZ-RhoGEF-overexpressing 3rd instar larval eye imaginal discs, determined by BrdU incorporation. (I) GMR-GAL4/+, (II) GMR-GAL4/UAS-DRhoGEF2, and (III) GMR-GAL4/UAS-mycPDZ-RhoGEF. (E) & (F) Phosphorylation of dPKB/dAkt in the 3rd instar larval eye imaginal discs with DRhoGEF2 (C) or PDZ-RhoGEF (D) overexpression. (TIF) [file pone.0152259.s003.tif]

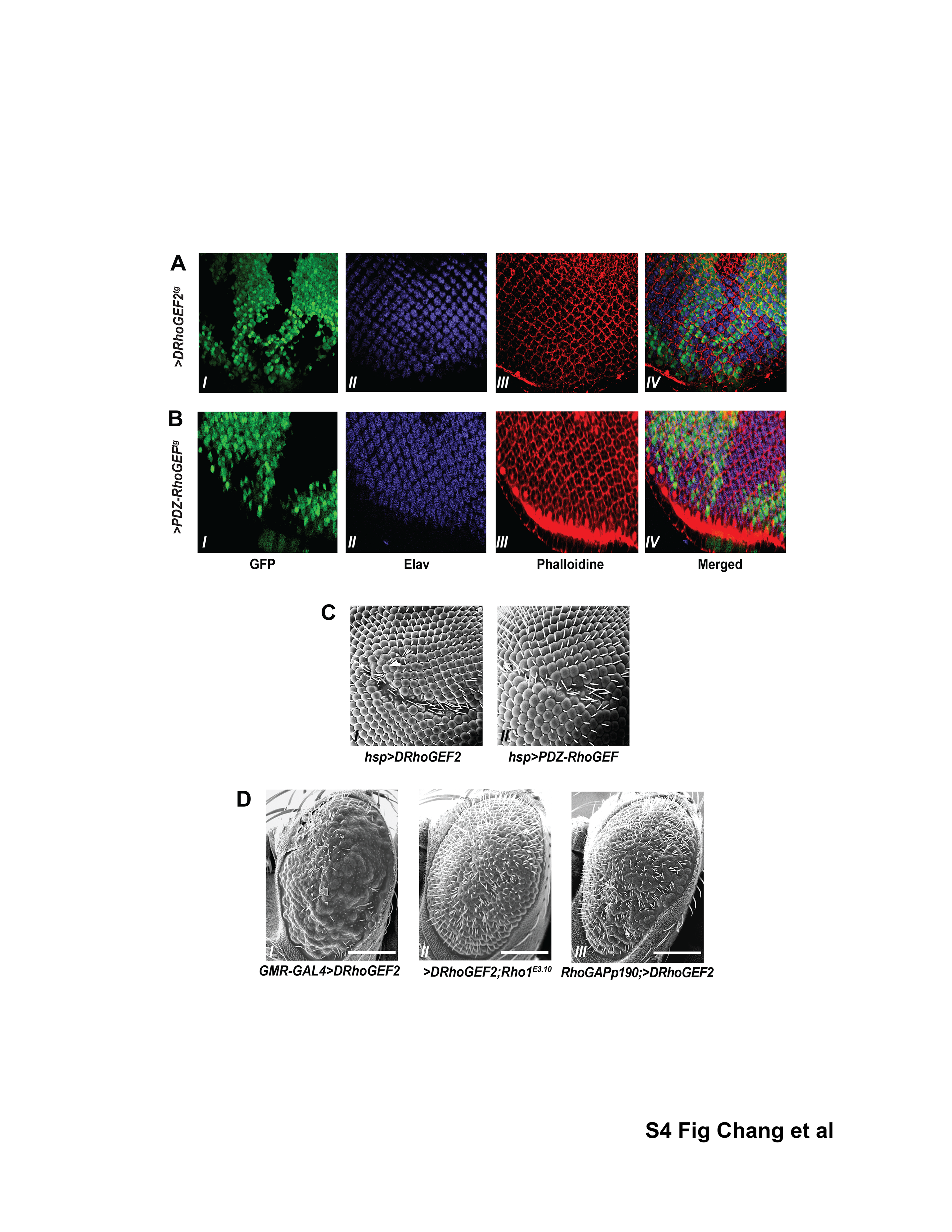

Supplement: S4 Fig — (A) & (B) Immunostaining of the post-mitotic neuronal cells with ectopic DRhoGEF2 (A) or PDZ-RhoGEF (B) expression induced by heat shock through mitotic recombination. (C) Scanning electronic micrographs of adult eyes from heat-induced recombination and gene expression. (I) hsflp;act,FRT,GAL4>UAS-GFP/UAS-DRhoGEF2 and (II) hsflp;act,FRT,GAL4>UAS-GFP/UAS-mycPDZ-RhoGEF. (D) Scanning electronic micrographs of adult fly eyes from GMR-GAL4>UAS-DRhoGEF2/CyO (I), GMR-GAL4>UAS-DRhoGEF2/RhoE3.10 (II), w67c23P{EPgy2}RhoGAPp190EY08765/+;GMR-GAL4>UAS-DRhoGEF2 (III). Scale bar = 200 μm. (TIF) [file pone.0152259.s004.tif]
