# Supplementary figures and images for: Correction: The Rho Guanine Nucleotide Exchange Factor DRhoGEF2 Is a Genetic Modifier of the PI3K Pathway in Drosophila
Source: PLoS One. 2021 May 20;16(5):e0252252. doi: 10.1371/journal.pone.0252252 (PMC8136627; doi:10.1371/journal.pone.0252252)

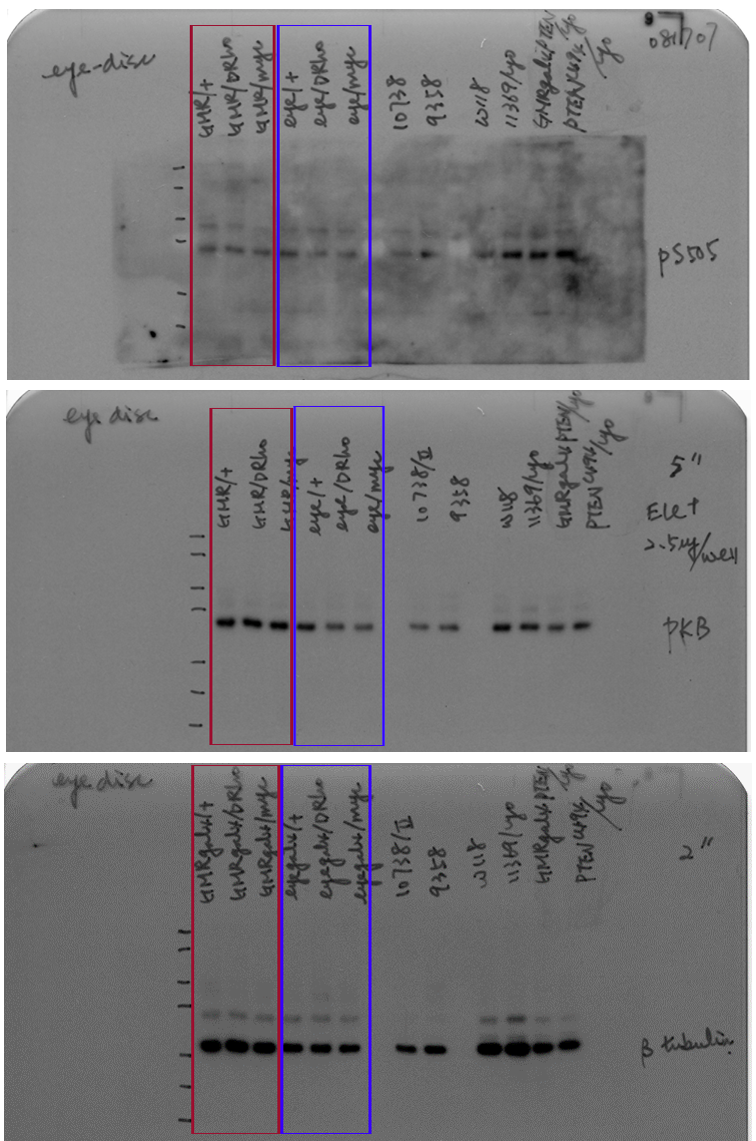

Supplement: S1 File — (TIF) [file pone.0252252.s001.tif]

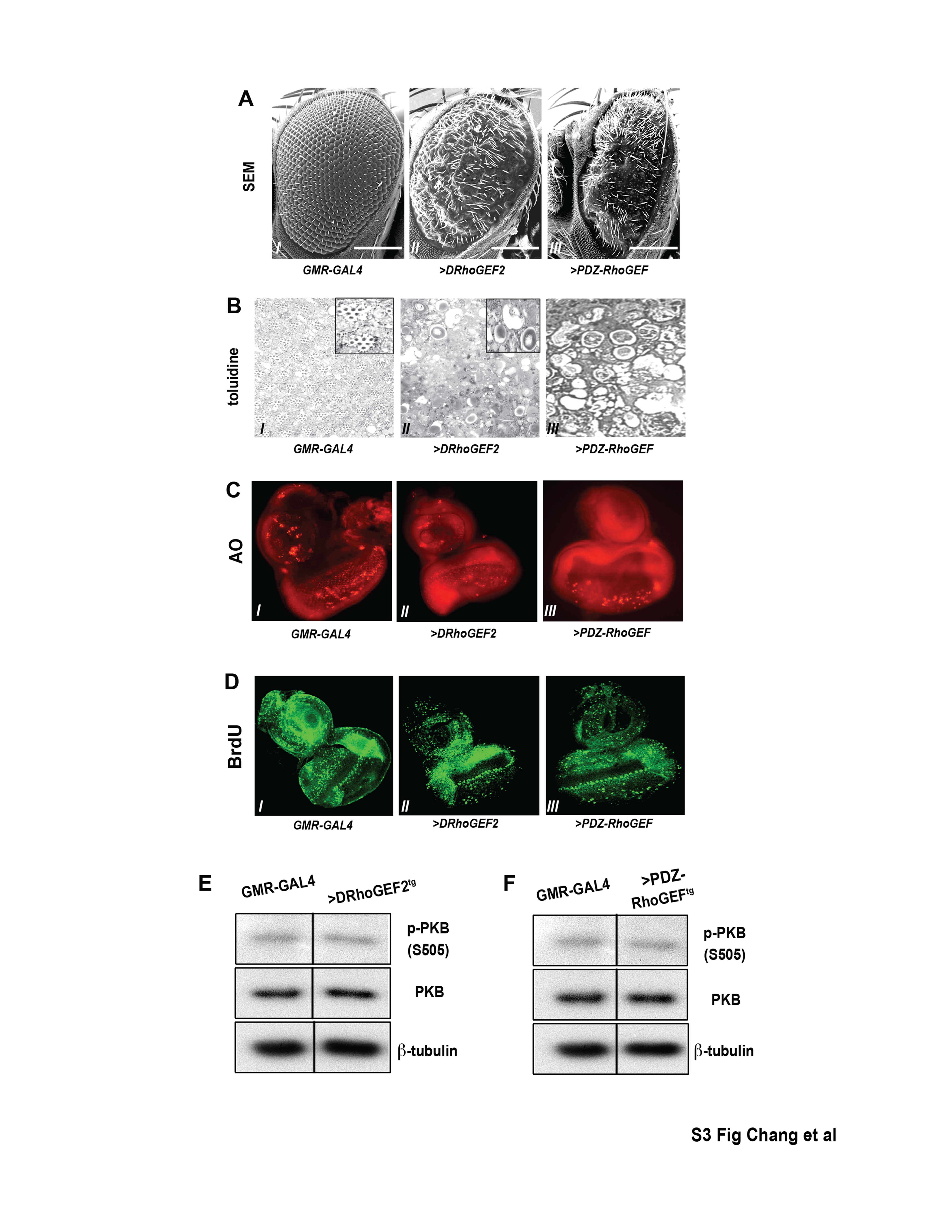

Supplement: S3 Fig — Labels in (A-D) indicate samples with the following genotypes: (I) GMR-GAL4/+, (II) GMR-GAL4/UAS-DRhoGEF2, and (III) GMR-GAL4/UAS-mycPDZ-RhoGEF. (A) Scanning electron micrographs of adult eyes with ectopic expression of DRhoGEF2 or mycPDZ-RhoGEF under the control of GMR-GAL4. Scale bar = 200 μm. (B) Toluidine blue-stained transverse sections of the adult eye with DRhoGEF2 or PDZ-RhoGEF overexpression. (C) Acridine orange (AO) staining in the 3rd instar larval eye imaginal discs with DRhoGEF2 or mycPDZ-RhoGEF overexpression. (D) Cell proliferation in DRhoGEF2- or PDZ-RhoGEF-overexpressing 3rd instar larval eye imaginal discs, determined by BrdU incorporation. (E, F) Phosphorylation of dPKB/dAkt in the 3rd instar larval eye imaginal discs of control GMR-GAL4 flies (E, F) or flies overexpressing DRhoGEF2 (E) or PDZ-RhoGEF (F). Results shown in panels E and F were obtained in the same western blot experiment for which underlying data are in S1 File of this Correction notice. (TIF) [file pone.0252252.s002.tif]
